# Case Report: Small intestinal metastatic breast cancer: A case report and literature review

**DOI:** 10.3389/fonc.2022.900832

**Published:** 2022-11-23

**Authors:** Yishan Li, Lianru Zhang, Huiping Yu, Xiaoyan Xin, Jian He, Yongzhong Yao, Baorui Liu, Rutian Li, Li Xie

**Affiliations:** ^1^ The Comprehensive Cancer Centre of Nanjing Drum Tower Hospital, The Affiliated Hospital of Nanjing University Medical School, Nanjing, China; ^2^ Department of Pathology, Nanjing Drum Tower Hospital, The Affiliated Hospital of Nanjing University Medical School, Nanjing, China; ^3^ Department of Radiology, Nanjing Drum Tower Hospital, The Affiliated Hospital of Nanjing University Medical School, Nanjing, China; ^4^ Departments of Nuclear Medicine, Nanjing Drum Tower Hospital, The Affiliated Hospital of Nanjing University Medical School, Nanjing, China; ^5^ Department of General Surgery, Nanjing Drum Tower Hospital, The Affiliated Hospital of Nanjing University Medical School, Nanjing, China

**Keywords:** breast cancer, small intestinal metastasis, small intestinal obstruction, clinical diagnosis, driving genes

## Abstract

Breast cancer is considered a malignant tumor with the highest incidence among women and is prone to develop distant metastasis. Small intestinal metastasis of breast cancer, however, is relatively rare. This case report describes a 49-year-old Chinese female patient who presented with small intestinal obstruction and was diagnosed with lobular breast cancer with small intestinal and contralateral breast metastasis. Clinical manifestations, clinicopathological features and potential mechanisms of metastasis, along with diagnosis and treatment, are discussed with a review of the relevant literature. Although small intestinal metastasis is rare in breast cancer, we should keep high alert on the possibility of gastrointestinal metastasis when treating lobular breast cancer patients.

## Introduction

The incidence of malignant small intestinal tumors, which account for approximately 2% of all gastrointestinal malignancies, is extremely low. Small intestinal malignancies occur more often among the North American population (with an age-standardized incidence of 1.6/100,000 in men and 1.1/100,000 in women), especially among African Americans (2.7/100,000 in men and 2.1/100,000 in women), followed by the European and Oceanian populations, while African, Central, South American, and Asian populations have a lower incidence (1). Metastatic tumors of the small intestine are also rare, often arising from direct invasion or intraperitoneal seeding, as well as hematogenous metastasis from extra-abdominal primary tumors (2, 3). In a retrospective study conducted by Bento et al., only 29 small intestinal metastases were found among 104 gastrointestinal metastatic sites in 95 patients, with melanoma, lung, and breast cancer being the most common types of primary malignancies (4).

As revealed by the global estimates from the International Agency for Research on Cancer in 2020, breast cancer accounted for 11.7% of all new cancer cases, making it the malignant tumor with the highest incidence (5). Bone, liver, lung, central nervous system, pleura, and peritoneum are all common sites of distant metastases from primary breast cancer (6). However, single small intestinal metastases, especially those with small intestinal obstruction, are extremely rare (2). Herein, we describe a case of small intestinal obstruction caused by distant metastasis from lobular breast cancer and attempt to determine the molecular characteristics of the tumor.

## Case presentation

**Figure 1:**
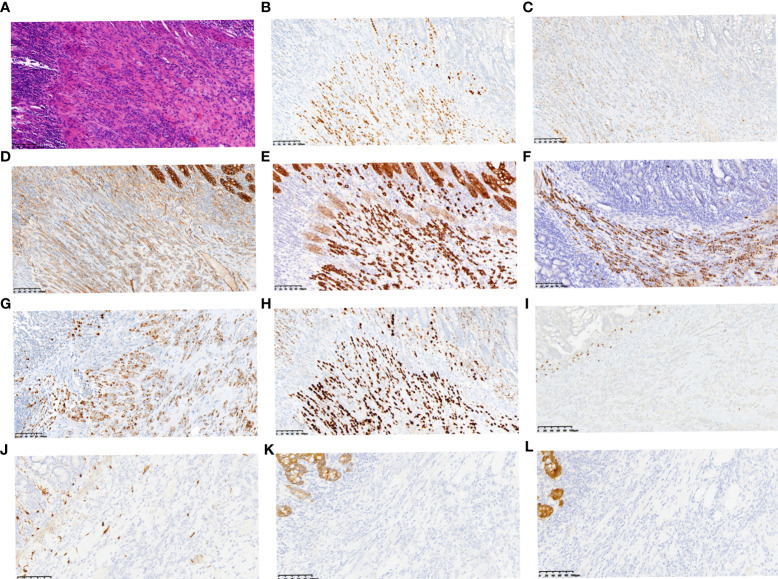
Representative histopathological results of small intestine metastasis. Small intestine metastasis was found to be diffuse infiltrating the submucosal muscularis propria and serosa **(A)** (HE staining, ×200, scale bars: 200 µm). Immunohistochemical staining revealed positive expression of ER **(B)** and PR **(C)**. Besides, p120 catenin was cytoplasmically positive **(D)**. CK **(E)**, CK7 **(F)**, Mammaglobin **(G)** and GATA3 **(H)** were all found to be strongly positive, while PAX8 **(I)**, S100 **(J)**, CK20 **(K)** and Villin **(L)** were weakly expressed or negative. (immunohistochemical staining, ×200, scale bars: 200 µm).

The patient was a 49-year-old Chinese woman who presented with upper abdominal pain accompanied by abdominal distention and vomiting for one day. Her sister was diagnosed with breast cancer at 45 years of age. Physical examination of the patient revealed abdominal distension with active bowel sounds and upper abdominal tenderness but no rebound tenderness or other abnormalities. Abdominal computed tomography (CT) revealed a thickened partial wall of the small intestine in the left lower abdomen, above which there was significant intestinal obstruction (**Figure S1**). Laparoscopic exploration surgery was performed under general anesthesia on November 4, 2020. Large amounts of ascites were observed during the operation, along with an obvious contracting segment of the intestine approximately 20 cm from the ileocecal region, while the proximal intestine expanded significantly with heavy edema and thickening of the intestinal wall. The patient underwent partial resection of the small intestine, and the specimen was sent for pathological examination. Postoperative pathology showed that the tumor was 1×0.8×0.6 cm in size, and microscopic examination results indicated invasive or metastatic carcinoma that may have originated from the breast, lung, or ovary. Tumor tissues were found to diffuse infiltrate into the submucosal muscularis propria and serosa, but no specific nerve or vascular infiltration nor residual tumor tissues were found on the upper or lower resection margins of the specimen (**Figure 1A**). Three lymph nodes adjacent to the intestine were examined, but metastasis was not observed. Ki67 was expressed in approximately 5% of tumor cells. The cells were strongly positive for estrogen receptor (ER) (**Figure 1B**) and progesterone receptor (PR) (**Figure 1C**). Besides, p120 catenin was cytoplasmically positive (**Figure 1D**). Cytokeratin (CK), CK7, mammaglobin, and GATA3 were found to be strongly positive (**Figures 1E–H**). PAX8 was weakly expressed (**Figure 1I**). No expression was observed for S100, CK20, or villin (**Figures 1J–L**).

**Figure 2:**
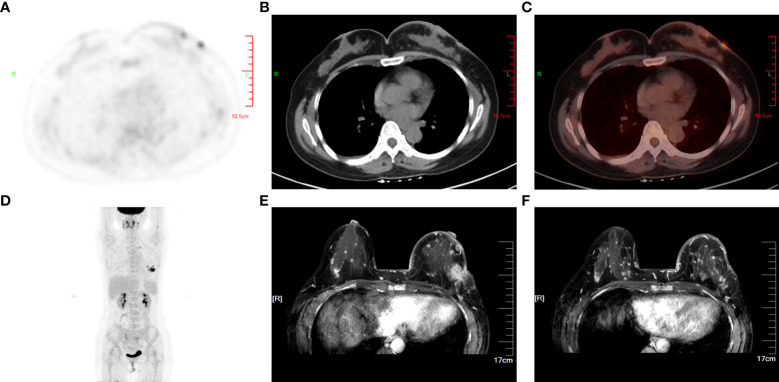
Primary lesion and invasive metastasis in the breast. PET/CT **(A–D)** revealed multiple masses with increased glucose metabolism in the lateral quadrant of the left breast as well as nodules with slightly increased glucose metabolism in the lateral quadrant of the right breast, which indicated aggressive invasion of the primary tumor on the left. MRI **(E, F)** in axial sections of the patient’s breast before chemotherapy showed the primary lesion in the left breast and multiple contralateral breast metastases.

**Figure 3:**
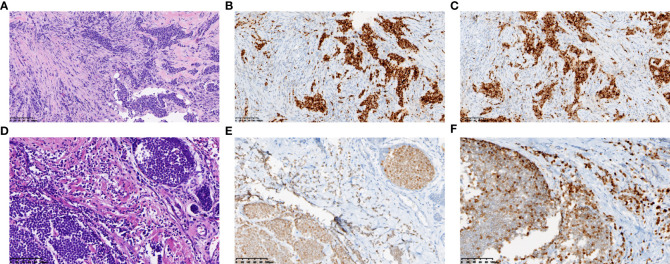
Representative histopathological results of the primary lesion in left breast. Needle biopsy revealed classic invasive lobular breast carcinoma **(A)**. **(A, D)** show tumor of the resected left breast before **(A)** and after **(D)** chemotherapy. (HE staining, ×200, scale bars: 200 µm) Immunohistochemical staining revealed strongly positive expression of ER **(B)** and PR **(C)** in the primary lesion, while medially or weakly expressed after chemotherapy (ER **E**, PR **F**). (immunohistochemical staining, ×200, scale bars: 200 µm).

Further examination of positron emission tomography (PET)/CT (**Figures 2A–D**) revealed multiple masses with increased glucose metabolism in the lateral quadrant of the left breast, as well as nodules with slightly increased glucose metabolism in the lateral quadrant of the right breast, indicating left breast cancer and possible malignancy in the right breast. Magnetic resonance examination (**Figures 2E, F**) of the patient showed asymmetrical bilateral breasts, with enlargement of the left breast and a lobulated mass approximately 3.6×2.5 cm in size, with high diffusion-weighted imaging (DWI) signal and perfusion curves in washout type. Interestingly, multiple abnormal nodular enhancements and lobulation were also observed in the bilateral breasts. The foci on both sides of the breast were characterized by a high DWI signal, with perfusion curves of the left breast in the washout type and perfusion curves of the right breast in the plateau type, implying that these small lesions in the bilateral breasts may be metastases. A needle biopsy was then performed, and pathological examination demonstrated that the mass was a classic invasive lobular breast carcinoma with World Health Organization (WHO) grade I and no carcinoma *in situ* (**Figure 3A**). Immunohistochemistry (IHC) results revealed that the breast cancer cells expressed human epidermal growth factor receptor 2 (HER2) (2+), and a lack of HER2 amplification was confirmed by fluorescence *in situ* hybridization. The tumor cells strongly expressed ER (90%, strongly positive) (**Figure 3B**) and PR (90%, strongly positive) (**Figure 3C**), with Ki67 expression in approximately 15% of the tumor cells.

When pathology, imaging results, and other assessments were analyzed in combination, the carcinoma was considered to be an invasive lobular left breast cancer with a pathologic TNM stage of cT4N0pM1 stage IV and metastasis to the small intestine and right breast. Although this was a case of stage IV breast cancer, the small intestinal metastasis had already been resected, and the malignant tumor sites were restricted to the breasts. Thus, surgical resection of the bilateral breasts was still possible after chemotherapy. The patient underwent six cycles of chemotherapy with paclitaxel liposomes combined with carboplatin from December 20, 2020, to March 25, 2021. Reexamination of the second and fourth cycles indicated shrinkage of the lesion in the left breast and improved condition of the superficial skin on the tumor; the curative effect was considered as partial remission. The patient underwent a bilateral simple mastectomy on May 4, 2021. Postoperative pathology suggested a 2.8×2.5×2-cm tumor bed in the lateral left papilla (**Figure 3D**). The adverse reactions were mild, and the Miller-Payne grade was 2. The cancer cells expressed HER2 (1+), ER (80%, medium to strong positivity) (**Figure 3E**), PR (60%, medium positivity) (**Figure 3F**), and Ki67 in the hotspots of approximately 5% of the cells. Multiple scattered small cancer lesions ranging from 0.05 cm to 0.2 cm in size were also found in the right breast, all of which were classic invasive lobular carcinomas (ILCs) with WHO grade I, expressing ER (90%, medium to strong positivity) and PR (weak positivity) but negative for HER2 expression. To inhibit ovarian function, the patient was prescribed endocrine therapy with the aromatase inhibitor anastrozole in combination with gonadotropin-releasing hormone.

**Figure 4:**
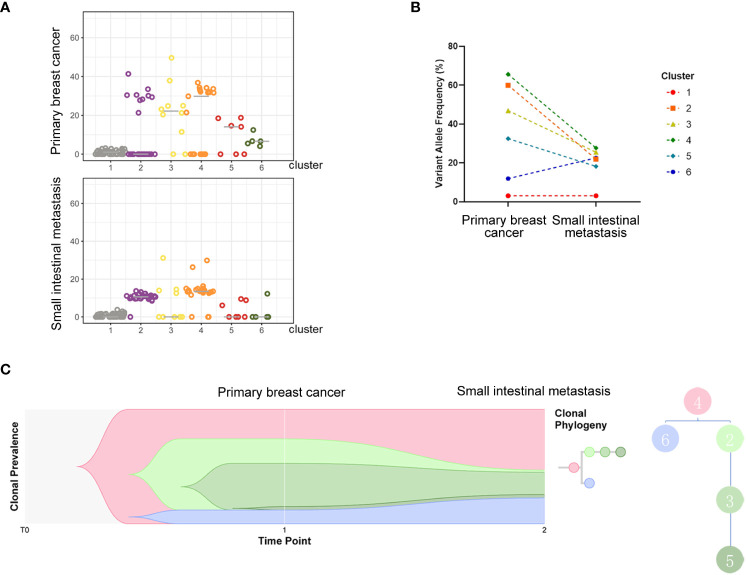
Analysis of subclones indicating evolutionary relations of clusters. All detected mutations of the two samples were clustered into 6 clones. The variant allele fraction (VAF) distribution of each detected mutation in different samples is shown in **(A)**, and changes in the average VAF of detected mutations between subclones of the two samples are exhibited in the charts of **(B)**. **(C)** indicates the evolutionary relations of clusters.

Next-generation sequencing analysis of tumor tissue from small intestinal metastasis and breast biopsy demonstrated that the driving genes of the primary lesion included mutations in *PIK3CA*, *CDH1*, and *AKT1*, as well as amplification of *CCND1* and *FGFR1*, which are common driving genes of breast cancer. Primary and metastatic tumors harbored relatively consistent driving genes because they shared 23 gene mutations, including *PIK3CA* and *CDH1* mutations and *CCND1* and *FGF19* amplifications (Tables S1 and S2). Metastatic tumors had higher mutation loads (58 versus 36 mutations) and higher levels of characteristic somatic cellular mutations (57 versus 39 mutations) than primary tumors. Panorama with somatic copy number variations exhibited common copy number variations, with minor differences in the genome between the two tumor lesions (**Figure S2**). The PyClone algorithm (7) was employed to cluster mutations in the primary tumor, and six clusters were identified. Cluster number 4, which contains genes such as *ARID1A*, *BCL9*, *BRWD1*, *CCDC116*, *CPD*, *CSNK1E*, *CYP1A1*, and *ERC1*, was found to be the ancestral subclone when clonal evolutionary relationships were analyzed (**Figure 4**). GO and KEGG pathway enrichment analyses were then employed to explore the functions of the differentially expressed genes and different pathways. Most genes in the metastatic lesions were enriched in the regulation of circadian rhythm (**Figure S3A**), whereas in the primary lesions, most differentially expressed genes were enriched in protein localization to the M-band (**Figure S3B**). In addition, metastatic lesions of the small intestine showed significant enrichment in cancer pathways (**Figure S3C**), whereas osteoclast differentiation was the most enriched in the primary breast lesion (**Figure S3D**).

## Discussion

It has long been accepted that the low incidence of tumors in the small intestine is due to a sterile state, consistent peristalsis, and the protective effects of mucosal immunity (8). Metastatic tumors of the small intestine are even rarer, with the majority occurring as advanced malignant tumors with multiple distant metastases. Small intestinal metastases mainly originate from gastrointestinal tumors and peritoneal carcinoma, followed by melanoma and breast or lung cancer (2, 3, 9). The ileum was the most common site of metastasis (9). In addition to intraperitoneal implantation, intestinal metastases also arise from hematogenous mechanisms (2, 3). With regard to clinical manifestations, patients often have gastrointestinal bleeding; intestinal obstruction; perforation symptoms such as abdominal pain, distention, and abdominal mass; nausea; and vomiting, among which abdominal pain is especially common (9, 10).

In terms of clinical imaging, endoscopic examination may not be the most suitable method for prompt diagnosis; in most cases, metastatic tumors often infiltrate the submucosa while leaving the mucosa seemingly complete (11). In our case, the metastatic tumor tissues of the small intestine invaded the submucosal muscularis propria and serosa. Further, detecting ILC on imaging is more difficult than detecting the more common invasive ductal carcinoma, especially on ^18^F-fluorodeoxyglucose (^18^F-FDG) PET/CT. ILCs usually demonstrate lower conspicuity because of their lower metabolic activity (12–14). However, there is still a possibility of detecting ILC using ^18^F-FDG PET/CT. In our case, ^18^F-FDG PET/CT revealed an increased glucose metabolism in the primary lesion. Other molecular imaging methods are needed to evaluate this malignancy and improve the possibility of detection.

McLemore et al. discovered that among 12,001 patients with metastases from primary breast cancer during the 15-year period from 1985 to 2000, only 53 metastases in 41 patients occurred in the gastrointestinal tract, and only 10 metastatic lesions were found in the small intestine. The study also revealed that among the 73 patients with primary breast cancer metastatic to the gastrointestinal tract or peritoneum, 12 (16%) were found to have metastases upon diagnosis, 15 (21%) were diagnosed with other diseases, and the rest were diagnosed when biopsy specimens were obtained through exploratory laparotomy or other operations. The average interval between the initial diagnosis and metastatic presentation was 7 years (15). Further, gastrointestinal metastases occur more often in patients with ILC. According to Idelevicha et al., among the 36 metastatic tumor patients who presented with intestinal obstruction between 1988 and 2005, 17 had metastases secondary to breast cancer, with lobular cancer being the only pathological type (16). In the present case, the patient first complained of symptoms of intestinal obstruction, including upper abdominal pain accompanied by abdominal distention and vomiting. In fact, a breast mass with skin involvement was overlooked by the patient. Metastatic tumors of the small intestine were diagnosed by pathological biopsy after partial resection of the small intestine. The transcription factor GATA3 (17, 18) and the highly expressed proteins mammaglobin and GCDFP15 (19), along with the combination of CK7, CK20, and villin (20), lead to the determination and diagnosis of primary breast cancer. IHC results of tumor cells from the intestinal mass revealed positive ER, PR, mammaglobin, and GATA3, as well as the CK7+/CK20-/villin- combination, all of which supported the conclusion of metastasis from breast cancer. Furthermore, genome analysis defined the breast origin of intestinal tumors, and clonal analysis proved that the intestinal metastasis evolved from the breast. Single intestinal metastases together with multiple tumor disseminations in the breast indicated that there may be special molecular events occurring in the intestinal metastatic tumor cells.

ILC is characterized by a mutation in the tumor suppressor gene *CDH1*, which encodes E-cadherin (21). Both frameshift mutations and aberrant promoter methylation of *CDH1* result in reduced or loss of E-cadherin expression. As long as E-cadherin plays a role in regulating cell adhesion and migration, *CDH1* mutation may promote metastasis of tumors and is thus closely related to the occurrence and progression of ILC (22). In addition, *PIK3CA* mutation keeps PI3K in a consistently active state and activates the downstream PI3K/AKT signaling pathway, thus reducing apoptosis and promoting tumor cell infiltration (23). Moreover, the key driving gene of breast cancer, *CCND1*, which is located on the amplicon of chromosome 11q13, mainly encodes cyclin D1, a protein that acts as a regulatory subunit and can activate CDK4/6 and phosphorylate Rb, thus facilitating cell entry from G1 to S phase. High expression of cyclin D1 results from the amplification of *CCND1*, which changes the course of the cell cycle and boosts the development of tumors. Additionally, overexpression of cyclin D1 is closely related to ER positivity and plays a role in the development of breast cancer because cyclin D1 acts directly on ER and activates it in an estrogen-independent manner (24, 25). Amplification of *FGFR1* is comparatively common in breast cancer, especially in ER-positive breast cancer, and is often associated with a poor prognosis, such as earlier relapse, lower survival rate, and resistance to endocrine therapy. *FGFR1* overexpression consistently stimulates the MAPK and PI3K/AKT/mTOR signaling pathways and enhances the progression of breast cancer by inducing tumor cell proliferation (26). It is also noteworthy that *CCND1* is commonly co-amplified with *FGFR1 (* 26), and *CCND1* is co-amplified with *FGF3*, *FGF4* and *FGF19* because of its colocalization with the 11q13 amplicon (27). Such co-amplification implies that both *CCND1* and the FGF/FGFR family play significant roles in the genesis and development of breast cancer and may synergistically interact with each other (27, 28). In our case of ER-positive breast cancer, both the primary tumor in the left breast and the small intestinal metastasis carried *CDH1* and *PIK3CA* mutations along with *CCND1* and *FGF19* amplification. Notably, the primary tumor in the left breast had a higher frequency of *CDH1* and *PIK3CA* mutations, and in addition to *FGFR1* amplification, there was *CCND1/FGF3/FGF4/FGF19* co-amplification. The underlying molecular mechanism indicated the aggressive character of the tumor cells, even though Ki67 expression was low.

Breast cancer metastasis involves multiple steps, including invasion of the basement membranes, intravasation into the vasculature, circulation, extravasation, and implantation into the target organ (29). Apart from initiating mutations of oncogenes, matrix cells in the microenvironment are always supportive of tumor cells gaining the ability to invade, preselect, and survive in the preferential target organs. The process of epithelial-to-mesenchymal transition (EMT), which is an irreplaceable step in triggering metastasis, involves the loss of epithelial markers, transdifferentiation into mesenchymal-like cells, and the acquisition of motile and invasive capacities (29). Transforming growth factor-β (TGF-β), a multifunctional cytokine generated by various histiocytes, promotes tumor progression through various mechanisms (30). TGF-β signaling in epithelial cells also induces the formation of a metastasis-favoring tumor microenvironment, as well as the expression of transcription factors incorporating Snail1/2, ZEB1/2, and HMGA2. As such, it can promote the EMT of tumors and inhibit the expression of adhesion proteins in epithelial cells while inducing the expression of mesenchymal proteins. In our case, two samples from the left breast primary lesion and the small intestinal metastasis were sent for second-generation sequencing, and all detected mutations were clustered into six groups of clones. When we compared the evolutionary trends of different clonal clusters between the two samples, cluster number 4 was found to be the ancestral clone because it decreased when evolving from a primary to a metastatic lesion, whereas only cluster number 6 presented an increasing trend. Hence, we concluded that there may be a correlation between metastasis and some of the genes in cluster 6, including *ANK2*, *CTDP1*, *GDF15*, *LTBP2*, and *SMG6*. The *LTBP2* gene, one of the four proteins in the LTBP family, plays critical roles in encoding the TGF-β protein, as well as in tumorigenesis, migration, and invasion (31). Research has shown that knockdown of *LTBP2* inhibits metastasis, invasion, and the EMT phenotype of tumor cells, thus inhibiting the genesis of thyroid carcinoma cells. In addition, increased expression of *LTBP2* was found to be related to a shorter overall survival in patients with gastric cancer (32). Further studies have revealed that *LTBP2* is highly expressed in both tissues and cell lines of gastric cancer, and silencing of the gene leads to decreased proliferation, migration, and invasion of gastric cancer cells. Another gene, *GDF15*, a divergent member of the bone morphogenetic protein subfamily of the TGF-β superfamily, also plays indispensable roles in the genesis, migration, and invasion of tumors (33, 34). *GDF15* levels have also been reported to be negatively related to the overall survival of colorectal cancer patients, and downregulation of *GDF15* suppresses EMT and metastasis in colorectal cancer cells. Based on the above-mentioned findings, we can presume that *LTBP2* and *GDF15* may be associated with small intestinal metastasis.

Systemic treatment should be administered first in patients with stage IV breast cancer. In our case, pathology of the patient’s breast biopsy after partial resection of the small intestine led to the diagnosis of stage IV lobular breast cancer. Since the carcinoma was characterized by a positive hormonal receptor and negative HER2 expression, endocrine therapy was the first choice. However, no lesion outside the breast was found on PET after resection of the small intestinal lesion, and the patient was also willing to undergo the operation after completing chemotherapy; therefore, chemotherapy combining paclitaxel liposomes and carboplatin was employed. After six cycles of chemotherapy, with an improved condition of the patient’s affected skin and reduced tumor size, a bilateral simple mastectomy was performed for surgical tumor reduction. Because the patient refused CDK4/6 inhibitors after the operation for economic reasons, the disease was controlled using the aromatase inhibitor anastrozole and ovarian function inhibition.

In conclusion, the patient presented with small intestinal obstruction symptoms was diagnosed with lobular breast cancer with small intestinal and multiple contralateral breast metastases. Chemotherapy combining paclitaxel liposomes and carboplatin was administered after palliative resection of the small intestinal lesion. The patient then underwent bilateral mastectomy, and endocrine therapy with an aromatase inhibitor was administered postoperatively. The same driving genes exist between primary and metastatic tumors; however, metastasis bears more tumor mutation loads. In addition, metastasis harbors more gene mutations related to invasive characteristics. Although small intestinal metastasis from breast cancer is comparatively rare, one should be aware of the possibility of gastrointestinal metastasis when treating patients with lobular breast cancer.

## Materials and methods

### Ethics statement

The patient provided written informed consent to be included in this case report.

### Whole-exome sequencing

For tumor tissue samples collected from primary and metastatic lesions, DNA was extracted using the FFPE kit (Promega). Sequencing libraries were constructed from native DNA using the xGen^®^ Exome Research Panel (Integrated DNA Technologies, Iowa, IA, USA) and the NEB Next Ultra DNA Library Prep Kit (Lot: NEB-0311611, NEB, UK) with a KAPA polymerase (KapaBiosystems, Wilmington, MA, USA) according to manufacturer’s instructions. Then whole-exome sequencing was performed using GeneSeq-2000 (Geneplus-Suzhou, Suzhou, China) with 100-bp paired-end reads. After removing the terminal adapter sequences and low-quality reads, the remaining high-quality reads were aligned with the human reference genome (hg19) using BWA MEM (v0.7.17–r1188). PCR duplicates were marked and removed with LocusCollector and Dedup, with realignment and recalibration performed with a Sentieon-genomics Realigner.

### Analysis of clonal population structures

The PyClone algorithm was employed to analyse the clonal population structures of samples from the primary and metastatic tumor, where six clusters were identified.

### Analysis of differentially expressed genes through GO and KEGG

For the analysis of GO (Gene Ontology) enrichment, we submitted the expression matrix of all genes and phenotypes of the samples to the GSEA software. Functional interpretation of the GO enrichment and KEGG (Kyoto Encyclopedia of Genes and Genomes) pathway enrichment analysis were carried out using the DAVID 9th database. The P-value <0.05 was considered statistically significant.

## Data availability statement

The original contributions presented in the study are included in the article/**Supplementary Material**. Further inquiries can be directed to the corresponding authors.

## Ethics statement

The patient provided written informed consent to be included in this case report.

## Author contributions

YL, LZ, and HY wrote and edited the manuscript. LZ, HY, XX, JH, and YY were involved in data collection and analysis. BL, RL, and LX contributed to conception and design of the study. All authors contributed to the article and approved the submitted version.

## Funding

This work was supported by grants from the National Natural Science Foundation of China (Grant No. 81803093).

## Conflict of interest

The authors declare that the research was conducted in the absence of any commercial or financial relationships that could be construed as a potential conflict of interest.

## Publisher’s note

All claims expressed in this article are solely those of the authors and do not necessarily represent those of their affiliated organizations, or those of the publisher, the editors and the reviewers. Any product that may be evaluated in this article, or claim that may be made by its manufacturer, is not guaranteed or endorsed by the publisher.
