# Supplementary material for: Case Report: Small intestinal metastatic breast cancer: A case report and literature review
Source: Front Oncol. 2022 Nov 23;12:900832. doi: 10.3389/fonc.2022.900832 (PMC9732937; doi:10.3389/fonc.2022.900832)
Supplement: Supplementary file 1 [file DataSheet_1.pdf]

frontiers

Supplementary Material

1 Supplementary Figures and Tables

1.1 Supplementary Figures Captions

Supplementary Figure 1. Small intestinal obstruction. Abdominal CT in axial (A) and sagittal (B) sections demonstrated significant intestinal obstruction (arrows).

Supplementary Figure 2. Distribution and panorama of somatic copy number variation. The panorama of somatic copy number variation (A) exhibits shared copy number variations and differences in the genome between the primary and metastatic lesions. The Venn diagram (B) compares the identical and characteristic somatic cellular mutations of primary and metastatic lesions.

Supplementary Figure 3. GO and KEGG pathway enrichment analysis. GO (Gene Ontology) enrichment analysis of differentially expressed genes in small intestinal metastatic lesions (A) and primary breast lesions (B) demonstrates significantly enriched molecular functions in GO categories. KEGG (Kyoto Encyclopedia of Genes and Genomes) pathway enrichment analysis illustrated the significantly enriched functional pathways of metastatic (C) and primary (D) lesions. The color and size of the dots represent the P value and number of genes enriched in the pathway, respectively.

1.2 Supplementary Tables

Supplementary Table 1. Representative shared point mutations, insertions and deletions of sample genes before chemotherapy

Genes	Transcripts	Base changes	Amino acid changes	Functional regions	Mutation frequency of primary tumor (%)	Mutation frequency of metastatic tumor (%)
CDH1	NM_004360.3	c.1147C>T	p.Q383*	EX9	43.9	13.2
PIK3CA	NM_006218.2	c.1624G>A	p.E542K	EX10	39.6	13.4
AKT1	NM_001014431.1	c.49G>A	p.E17K	EX3	2.1	-

Supplementary Table 2. Other representative shared structural variations of the two sample genes before chemotherapy

Genes	Transcripts	Variation type	Functional regions	Mutation frequency/copy coefficients	
				primary tumor	metastatic tumor
FGFR1	NM_023110.2	Amplification	8p11.23	1.94	-
CCND1	NM_053056.2	Amplification	11q13.3	3.42	1.84
FGF19	NM_005117.2	Amplification	11q13.3	3.43	1.81
FGF4	NM_002007.2	Amplification	11q13.3	3.97	-
FGF3	NM_005247.2	Amplification	11q13.3	3.95	-
