# Supplementary figures and images for: Case Report: Small intestinal metastatic breast cancer: A case report and literature review
Source: Front Oncol. 2022 Nov 23;12:900832. doi: 10.3389/fonc.2022.900832 (PMC9732937; doi:10.3389/fonc.2022.900832)

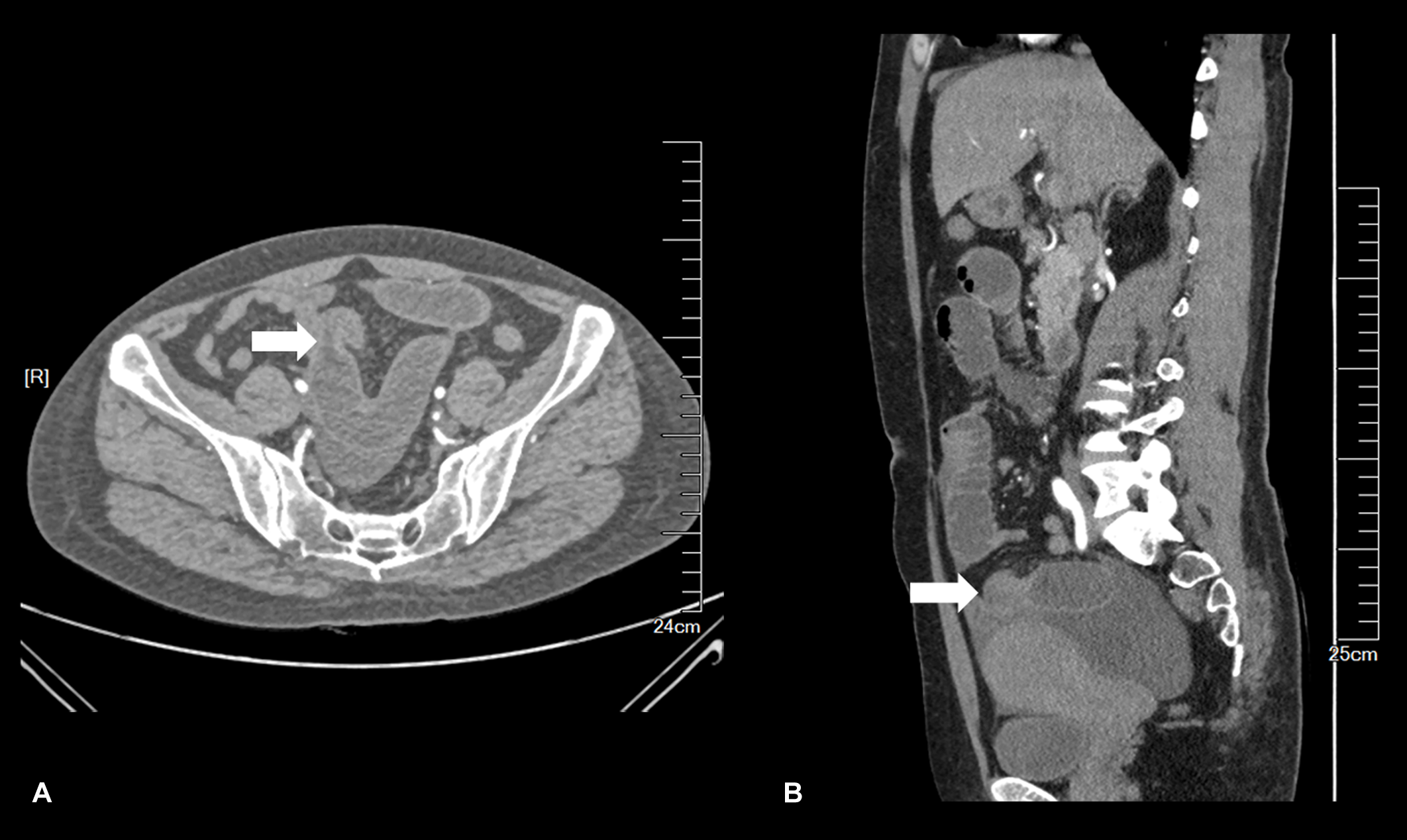

Supplement: Supplementary file 4 [file Image_1.tif]

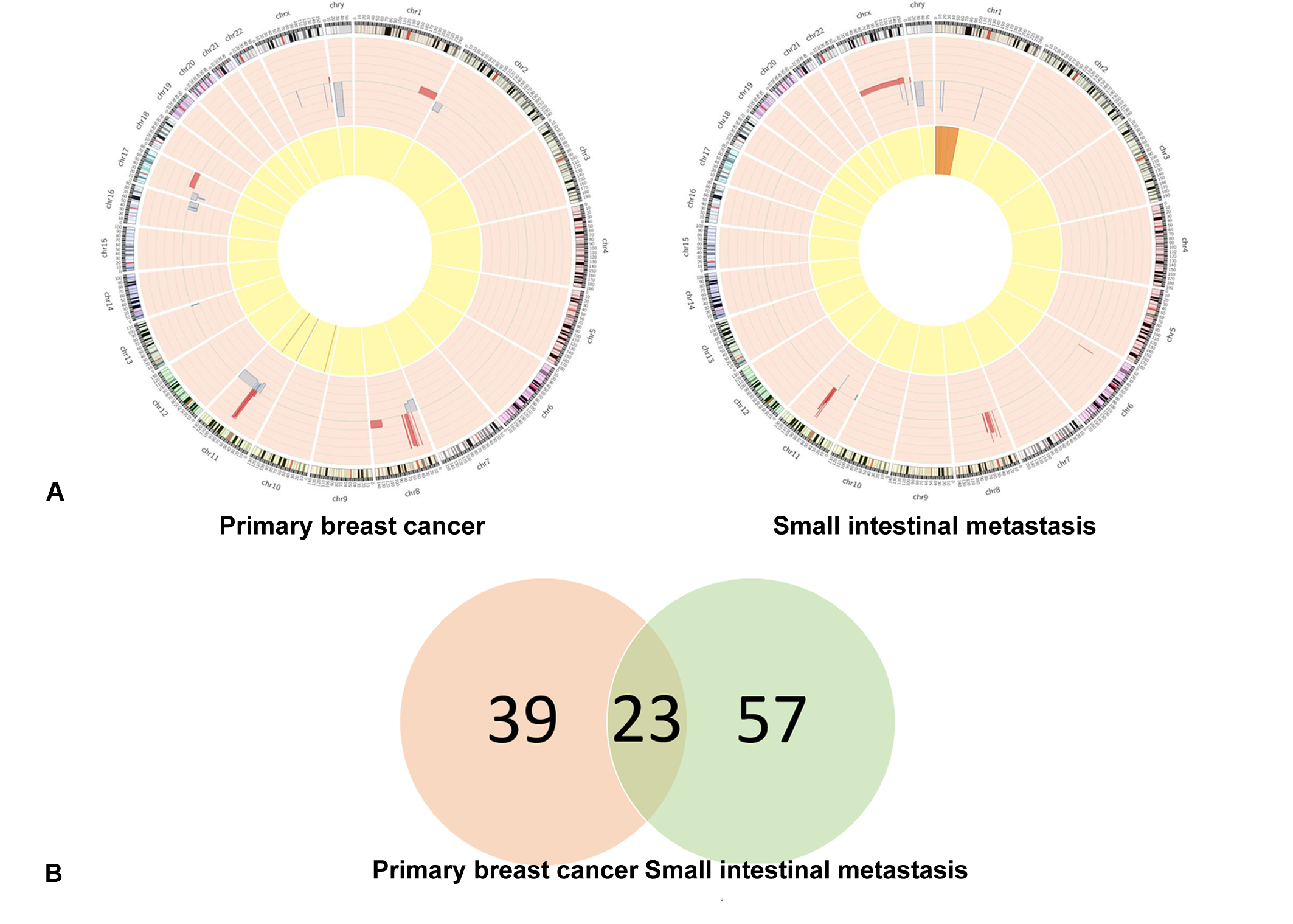

Supplement: Supplementary file 5 [file Image_2.tif]

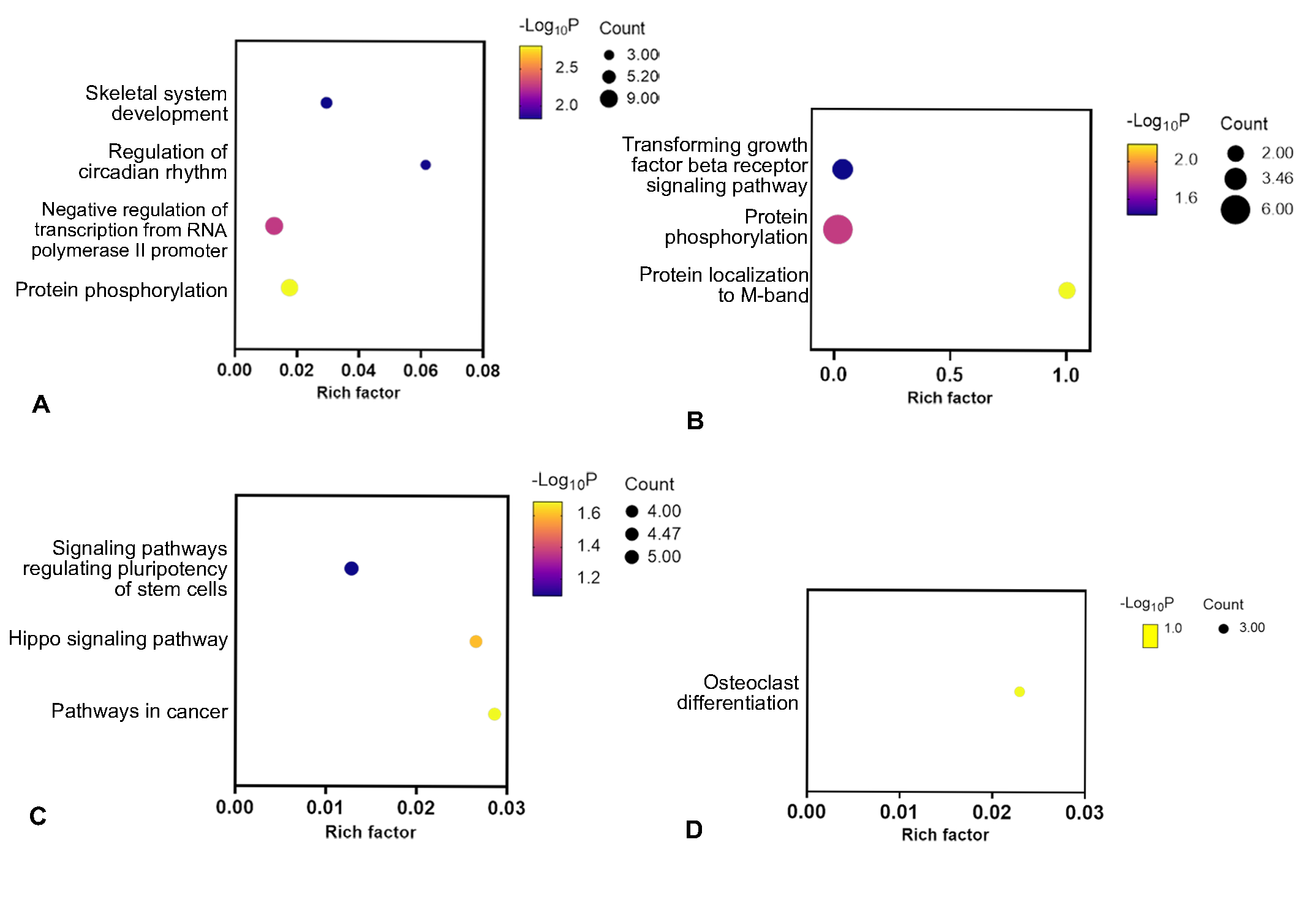

Supplement: Supplementary file 6 [file Image_3.tiff]
